# Supplementary material for: Associations of Lipids and Lipid-Lowering Drugs with Risk of Vascular Dementia: A Mendelian Randomization Study
Source: Nutrients. 2022 Dec 23;15(1):69. doi: 10.3390/nu15010069 (PMC9824558; doi:10.3390/nu15010069)
Supplement: Supplementary file 1 [file nutrients-15-00069-s001.zip › supplementary figure.pdf]

Supplementary Materials

Supplementary Figure S1A: Scatter plot showing the association of the SNP effects on HDL-C level against the SNP effects on vascular dementia;

Supplementary Figure S1B: Scatter plot showing the association of the SNP effects on LDL-C level against the SNP effects on vascular dementia;

Supplementary Figure S1C: Scatter plot showing the association of the SNP effects on TG levels against the SNP effects on vascular dementia;

Supplementary Figure S1D: Scatter plot showing the association of the SNP effects on apoA-I level against the SNP effects on vascular dementia;

Supplementary Figure S1E: Scatter plot showing the association of the SNP effects on the apoB level against the SNP effects on vascular dementia;

Supplementary Figure S2A: Leave-one-out permutation analysis of the causal association between HDL-C level and vascular dementia;

Supplementary Figure S2B: Leave-one-out permutation analysis of the causal association between LDL-C level and vascular dementia;

Supplementary Figure S2C: Leave-one-out permutation analysis of the causal association between TG level and vascular dementia;

Supplementary Figure S2D: Leave-one-out permutation analysis of the causal association between apoA-I level and vascular dementia;

Supplementary Figure S2E: Leave-one-out permutation analysis of the causal

association between apoB level and vascular dementia;

Supplementary Figure S3A: Scatter plot showing the association of the SNP effects on vascular dementia against the SNP effects on LDL-C level;

Supplementary Figure S3B: Scatter plot showing the association of the SNP effects on vascular dementia against the SNP effects on TG levels;

Supplementary Figure S3C: Scatter plot showing the association of the SNP effects on vascular dementia against the SNP effects on the apoA-I level;

Supplementary Figure S3D: Scatter plot showing the association of the SNP effects on vascular dementia against the SNP effects on the apoB level;

Supplementary Figure S4A: Leave-one-out permutation analysis of the causal association between vascular dementia and LDL-C level;

Supplementary Figure 4B. Leave-one-out permutation analysis of the causal association between vascular dementia and TG level;

Supplementary Figure S4C: Leave-one-out permutation analysis of the causal association between vascular dementia and apoA-I level;

Supplementary Figure S4D: Leave-one-out permutation analysis of the causal association between vascular dementia and apoB level;

Supplementary Figure S5A: Scatter plot showing the association of the SNP effect on LDL-C level mediated by the *HMGCR* gene against the SNP effects on vascular dementia;

Supplementary Figure S5B: Scatter plot showing the association of the SNP effect on LDL-C level mediated by the *PCSK9* gene against the SNP effects on vascular dementia;

Supplementary Figure S5C: Scatter plot showing the association of the SNP effect on LDL-C level mediated by the *NPC1L1* gene against the SNP effects on vascular dementia;

Supplementary Figure S5D: Scatter plot showing the association of the SNP effect on LDL-C level mediated by the *APOB* gene against the SNP effects on vascular dementia;

Supplementary Figure S6A: Leave-one-out permutation analysis of the causal association between LDL-C level mediated by the *HMGCR* gene and vascular dementia;

Supplementary Figure S6B: Leave-one-out permutation analysis of the causal association between LDL-C level mediated by the *PCSK9* gene and vascular dementia;

Supplementary Figure S6C: Leave-one-out permutation analysis of the causal association between LDL-C level mediated by the *NPC1L1* gene and vascular dementia;

Supplementary Figure S6D: Leave-one-out permutation analysis of the causal Supplementary association between LDL-C level mediated by the *APOB* gene and vascular dementia;

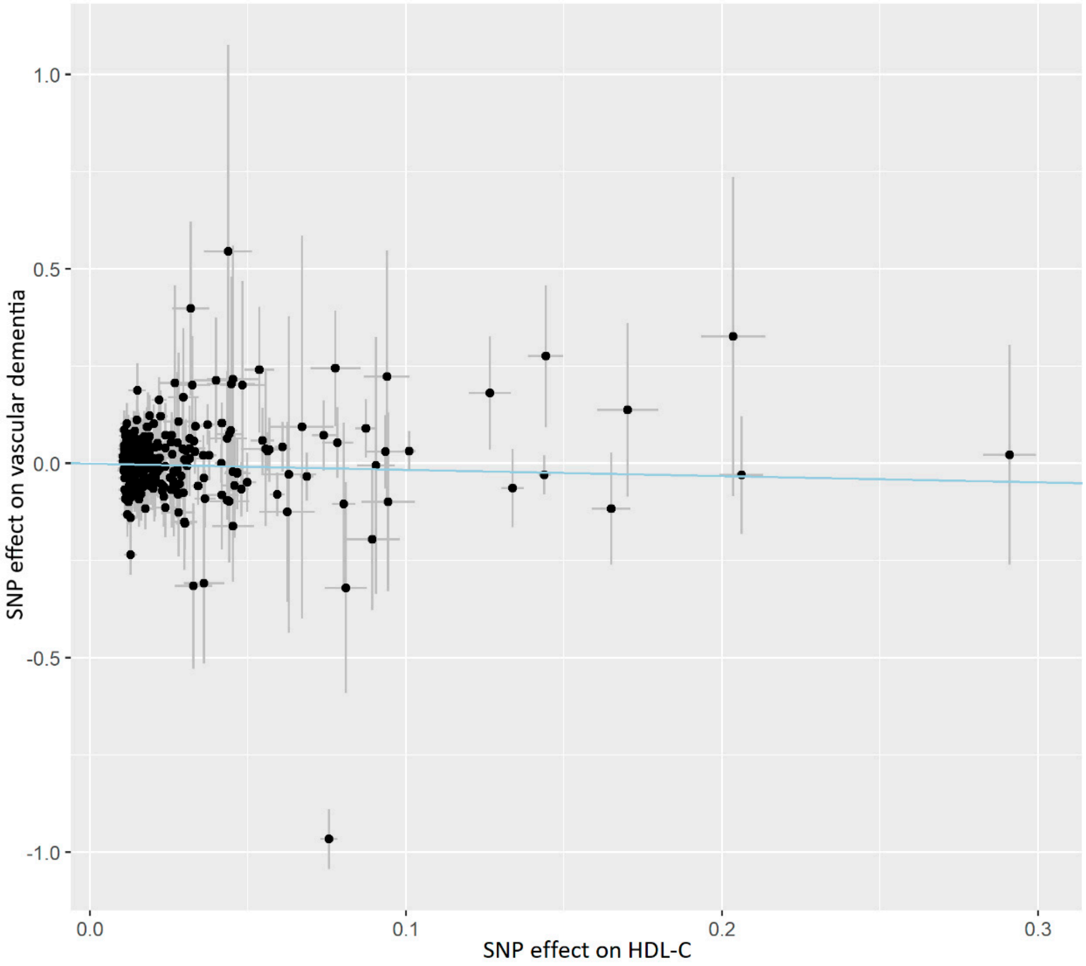

Supplementary Figure S1A. Scatter plot showing the association of the SNP effects on HDL-C level against the SNP effects on vascular dementia.

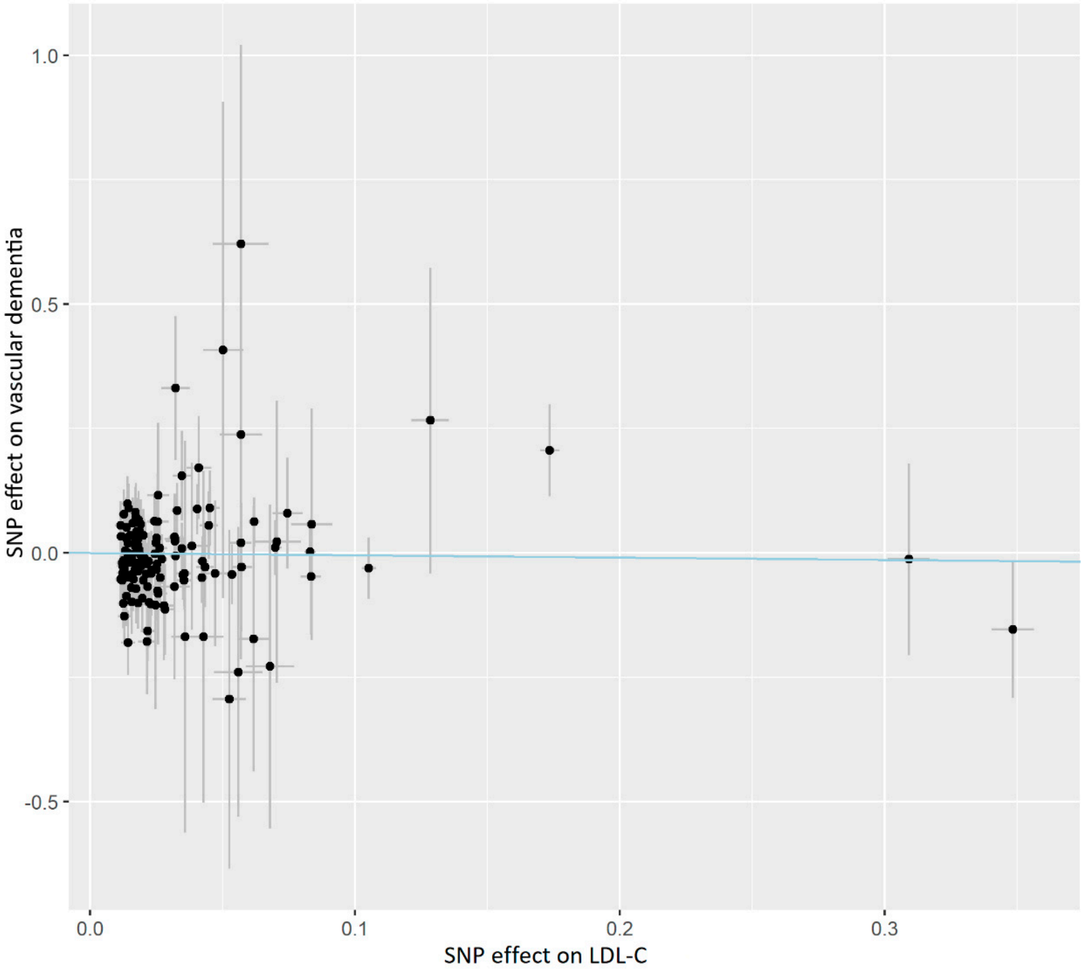

Supplementary Figure S1B. Scatter plot showing the association of the SNP effects on LDL-C level against the SNP effects on vascular dementia.

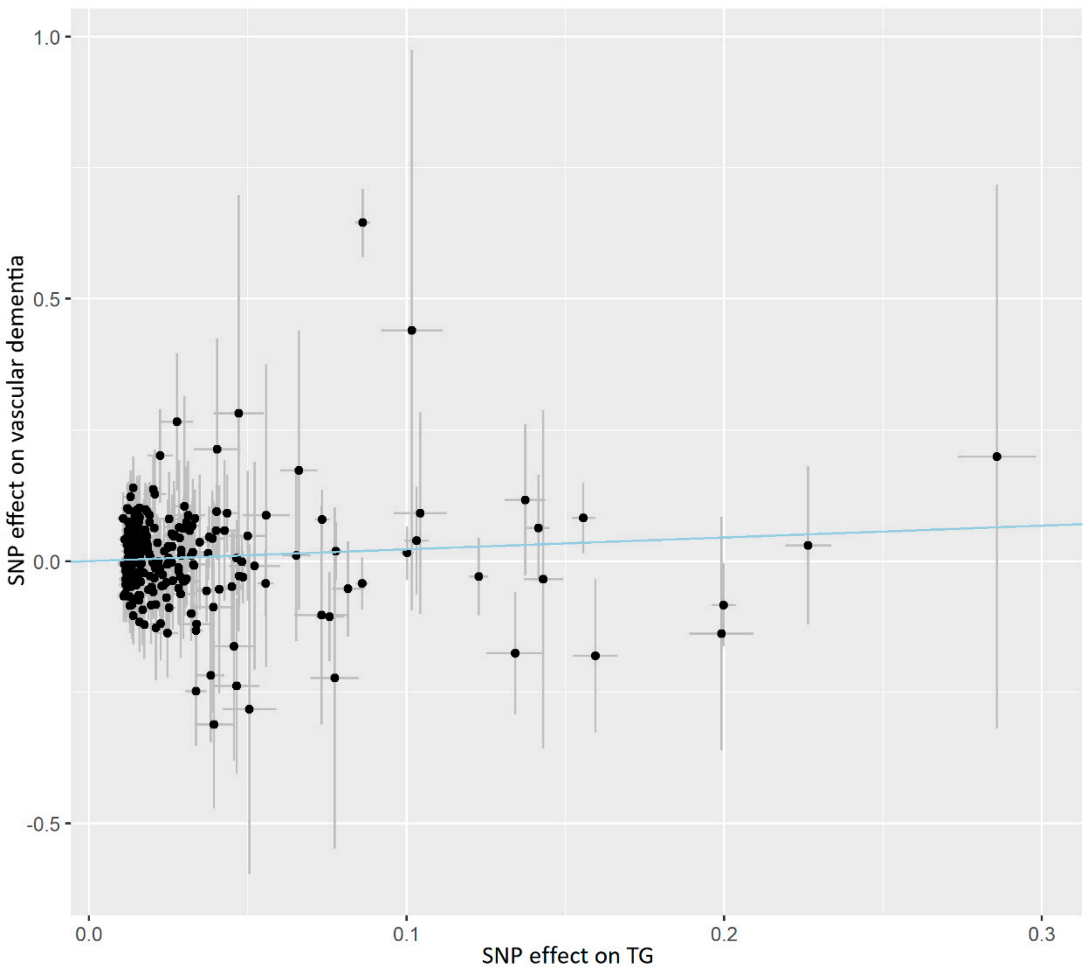

Supplementary Figure S1C. Scatter plot showing the association of the SNP effects on TG levels against the SNP effects on vascular dementia.

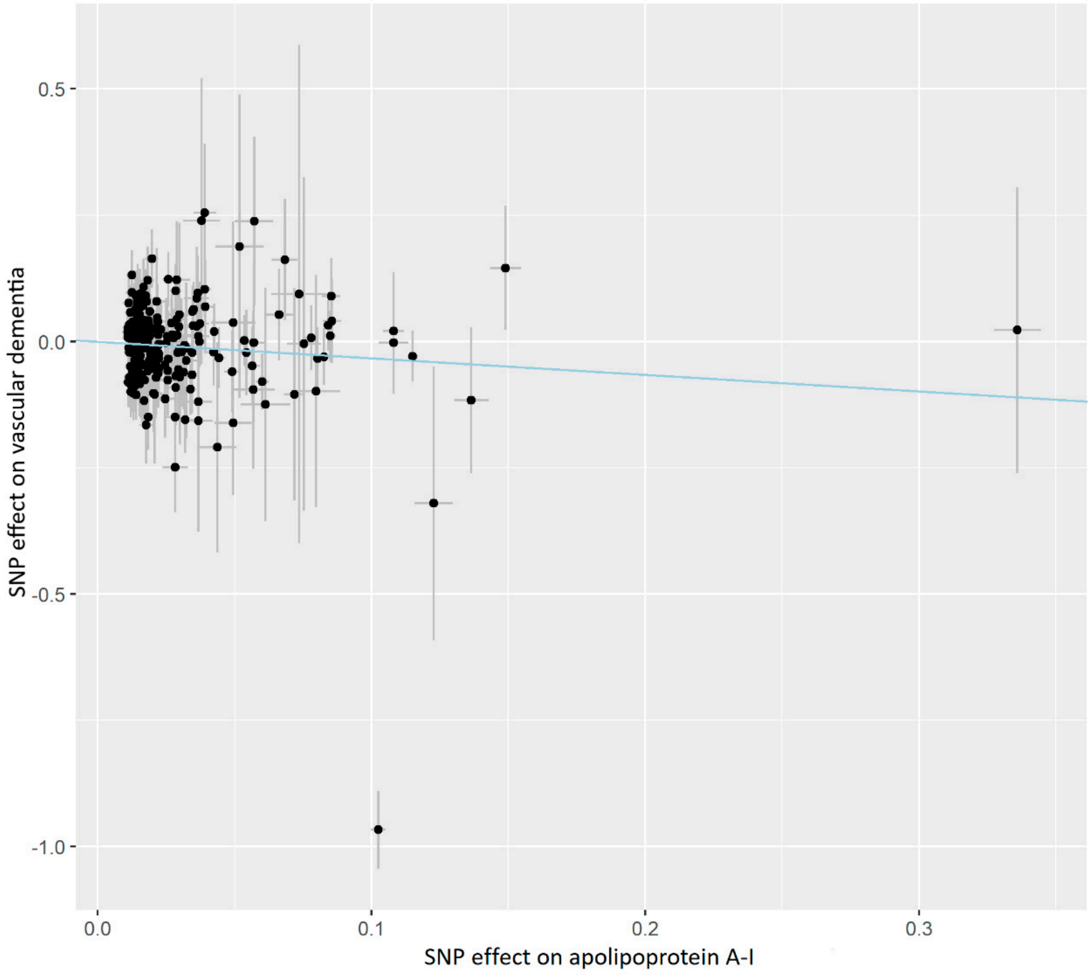

Supplementary Figure S1D. Scatter plot showing the association of the SNP effects on apoA-I level against the SNP effects on vascular dementia.

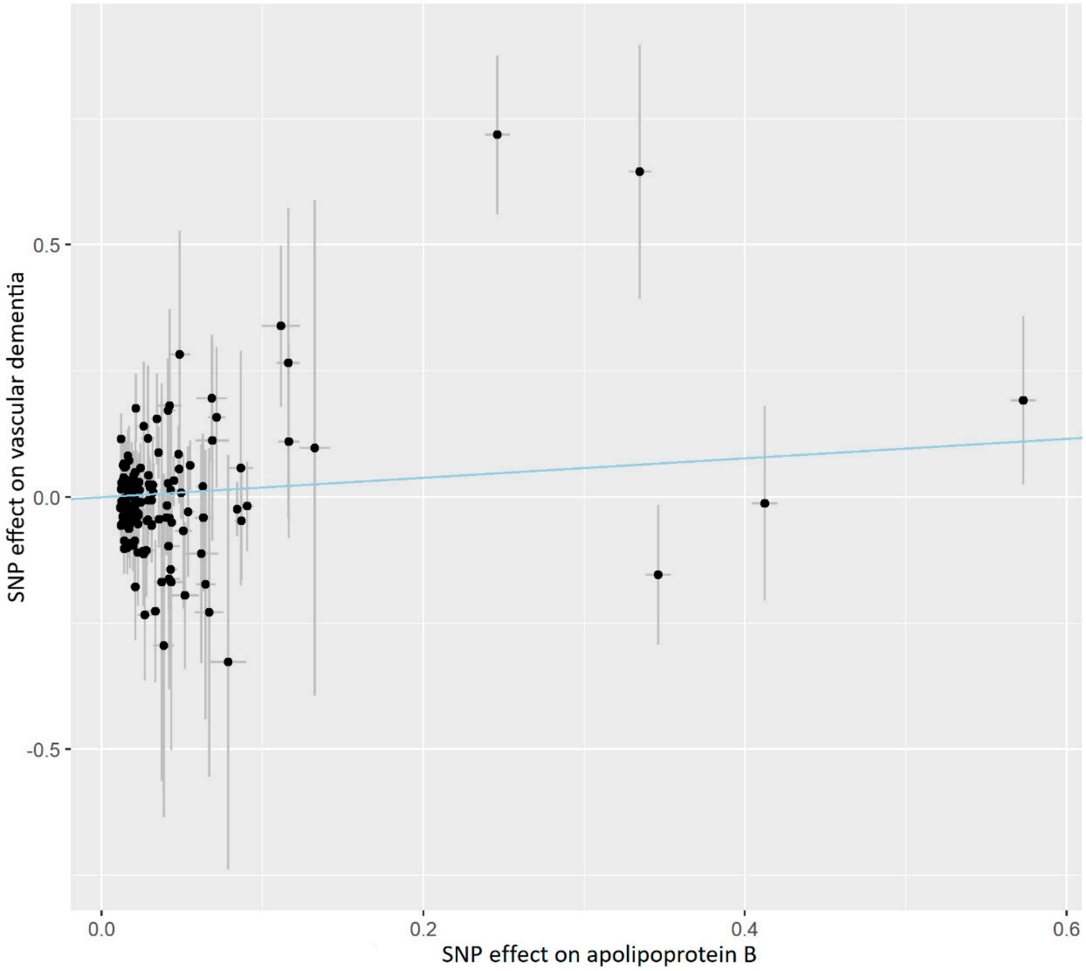

Supplementary Figure S1E. Scatter plot showing the association of the SNP effects on the apoB level against the SNP effects on vascular dementia.

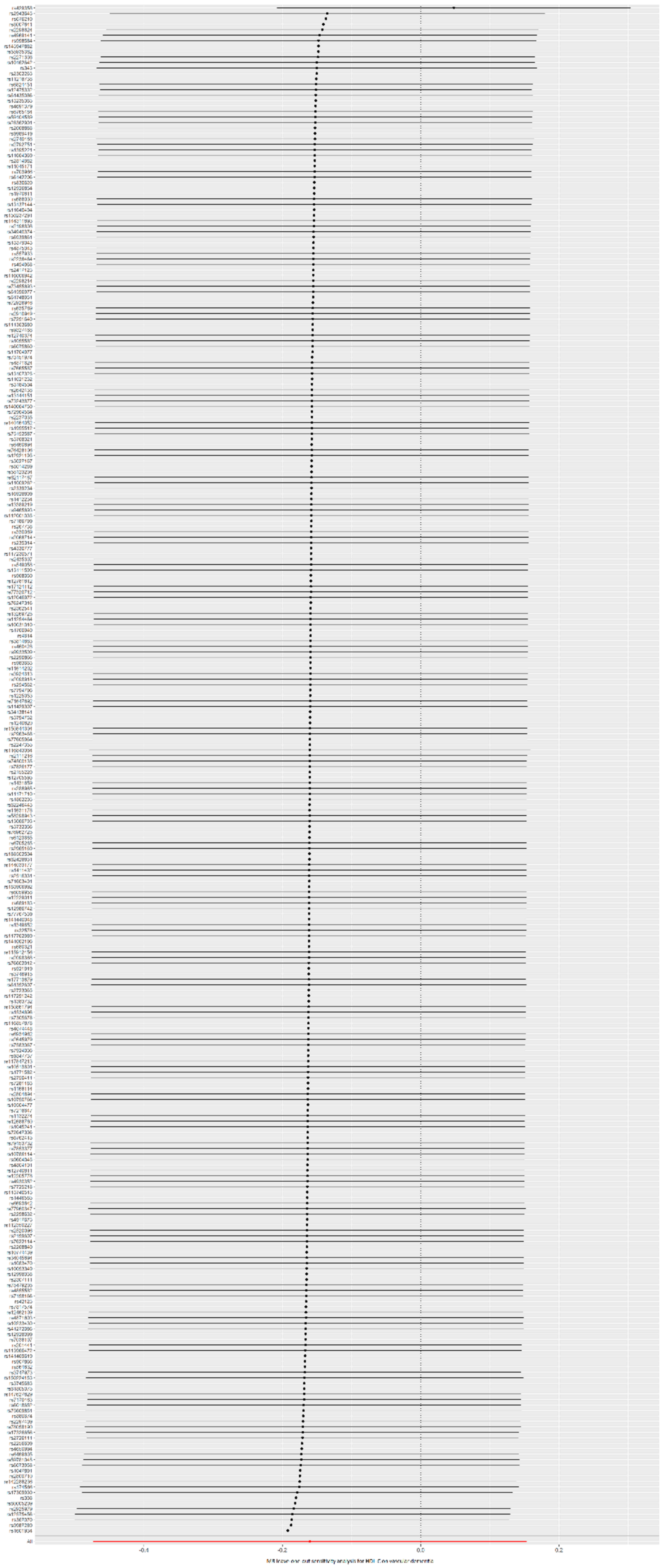

-0.4
-0.2
0.0
0.2
MR leave-one-out sensitivity analysis for HDL-C on vascular dementia

Supplementary Figure S2A. Leave-one-out permutation analysis of the causal association between HDL-C level and vascular dementia.

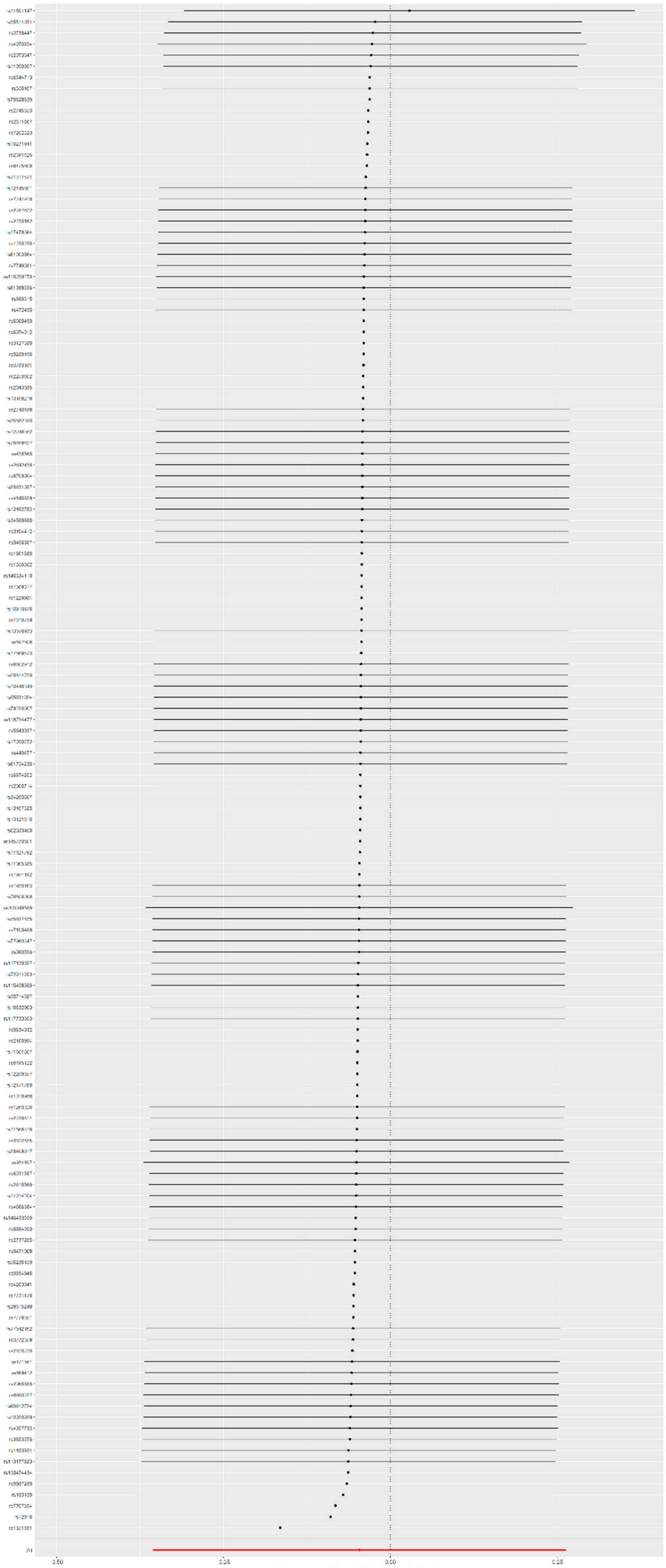

MR leave-one-out sensitivity analysis for LDL-C on vascular dementia

Supplementary Figure S2B. Leave-one-out permutation analysis of the causal association between LDL-C level and vascular dementia.

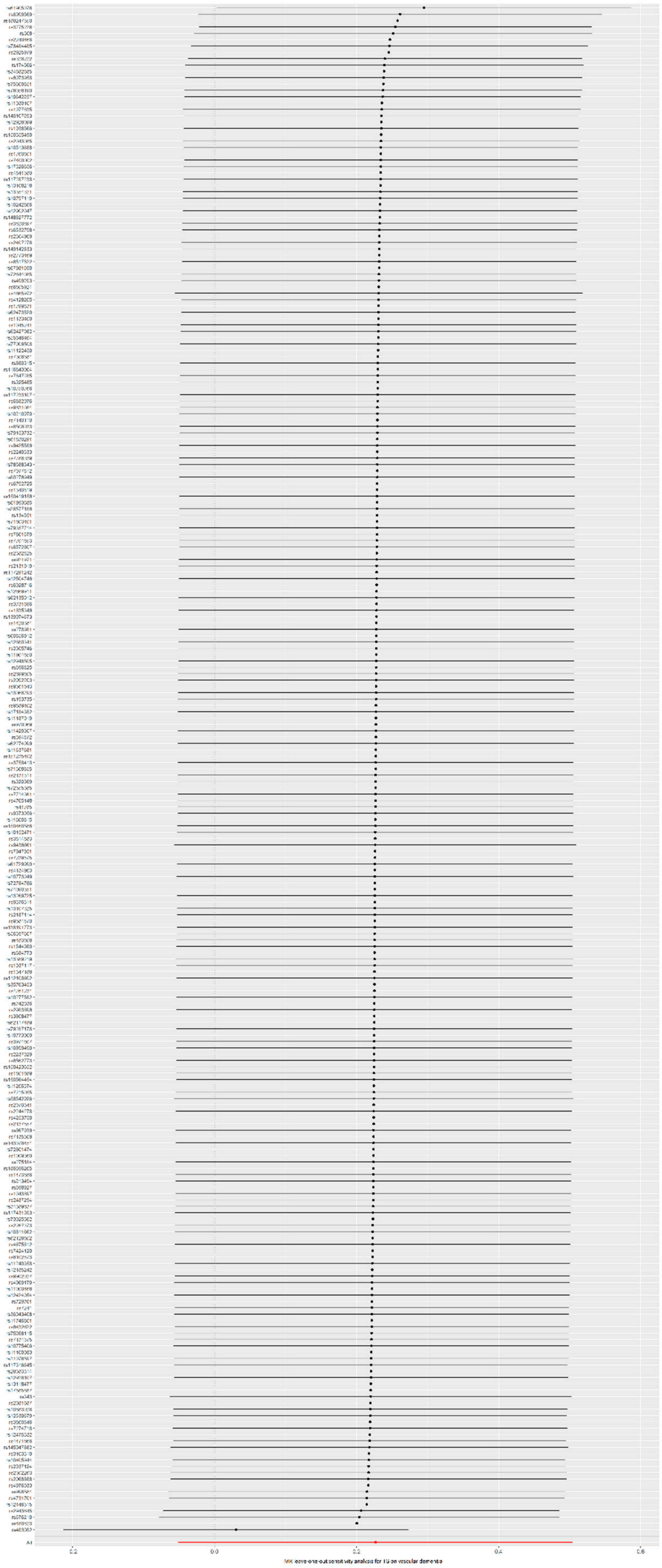
All
-0.2
0.0
0.2
0.4
0.6
MR leave-one-out sensitivity analysis for TG on vascular dementia

Supplementary Figure S2C. Leave-one-out permutation analysis of the causal association between TG level and vascular dementia.

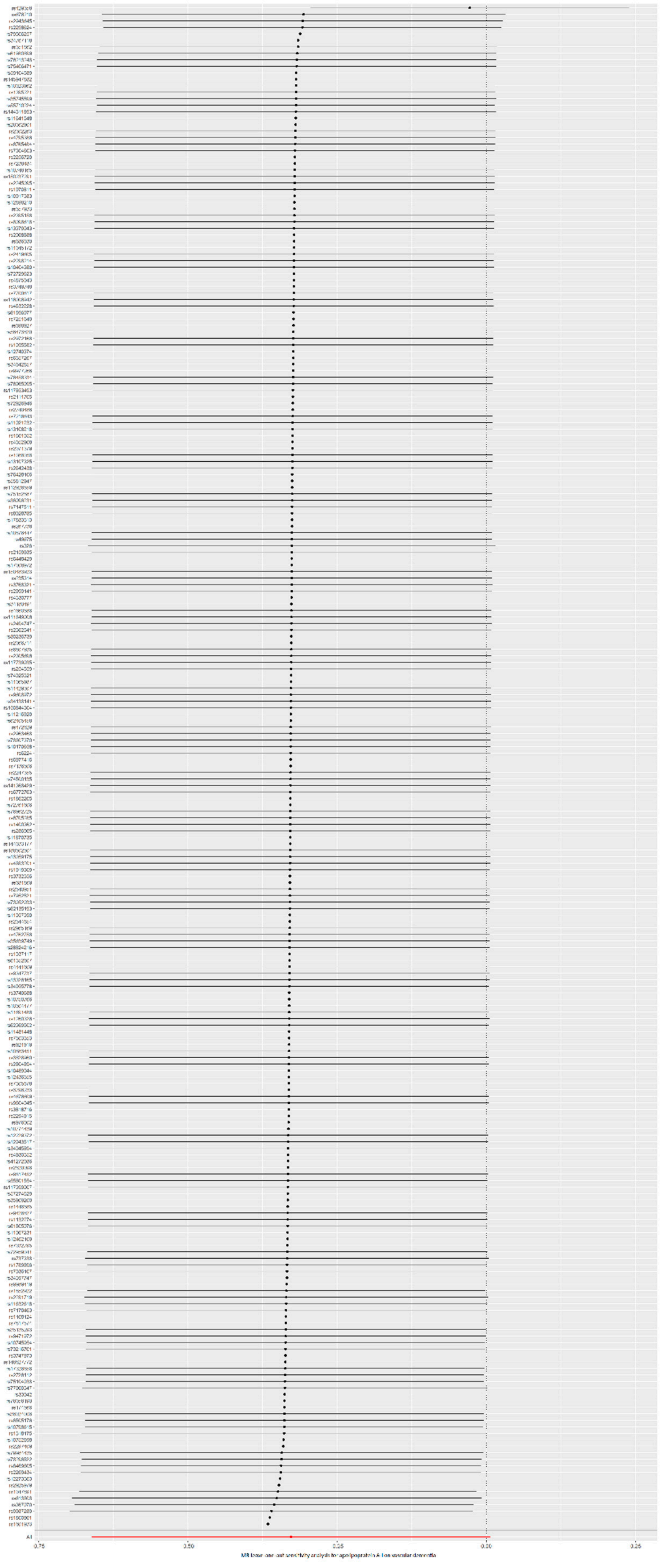
All
0.75
0.50
0.25
0.00
0.25
MR leave-one-out sensitivity analysis for apolipoprotein A-I on vascular dementia

Supplementary Figure S2D. Leave-one-out permutation analysis of the causal association between apoA-I level and vascular dementia.

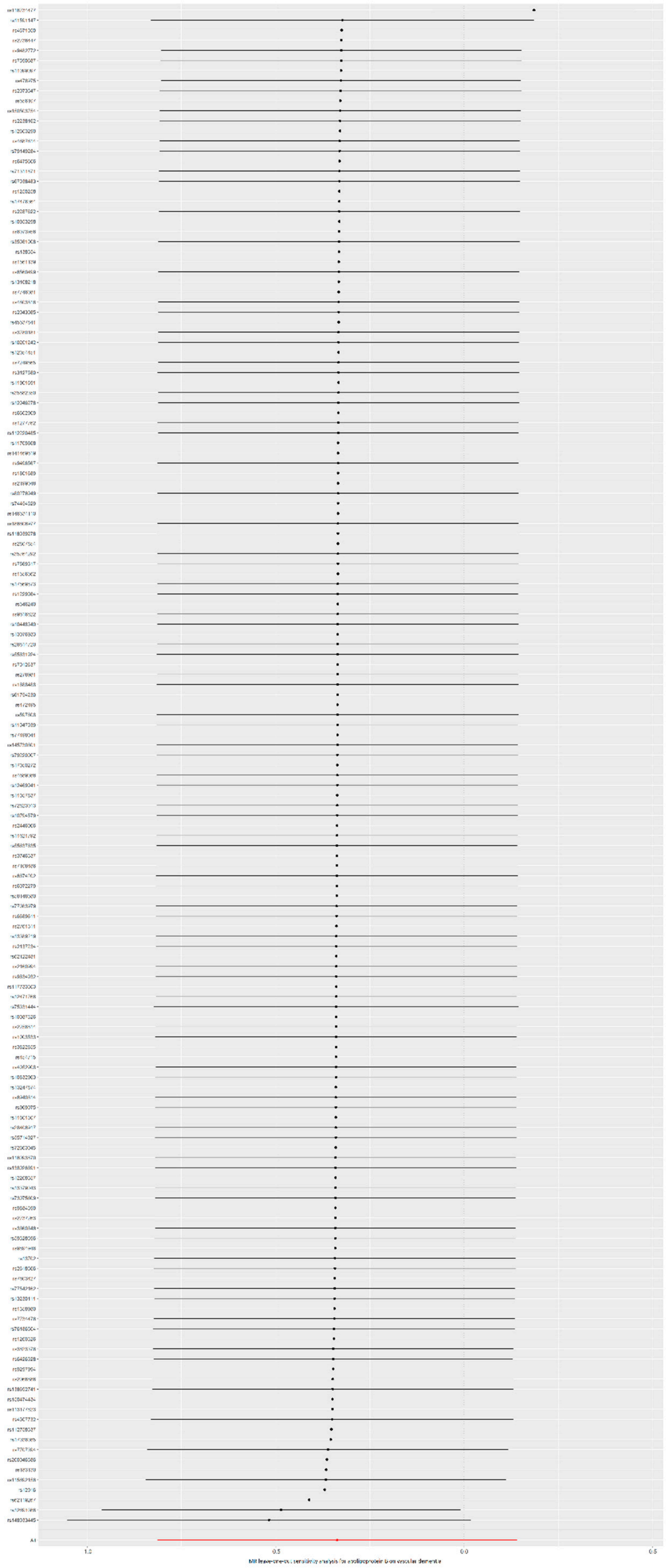
rs148033445
All
1.0
0.5
0.0
0.5
MR leave-one-out sensitivity analysis for apolipoprotein B on vascular dementia

Supplementary Figure S2E. Leave-one-out permutation analysis of the causal association between apoB level and vascular dementia.

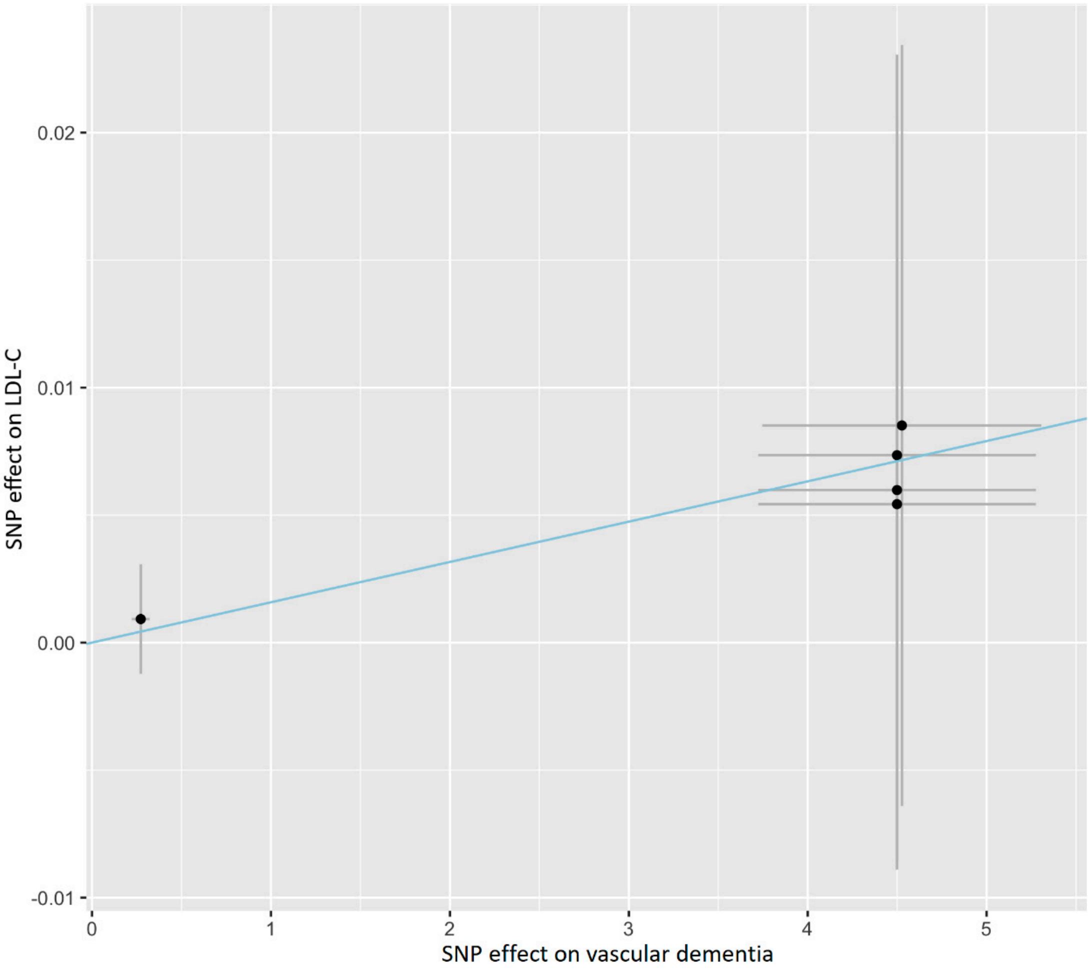

Supplementary Figure S3A. Scatter plot showing the association of the SNP effects on vascular dementia against the SNP effects on LDL-C level.

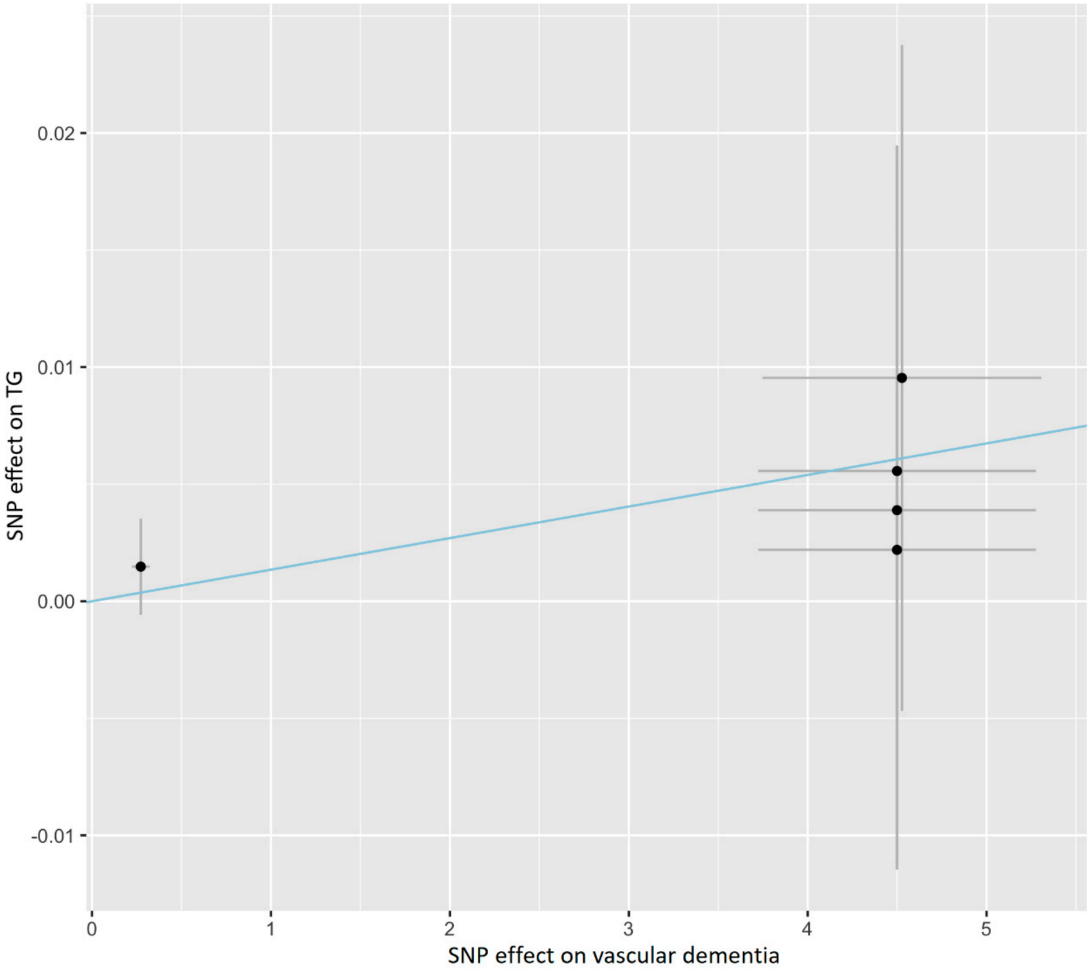

Supplementary Figure S3B. Scatter plot showing the association of the SNP effects on vascular dementia against the SNP effects on TG level.

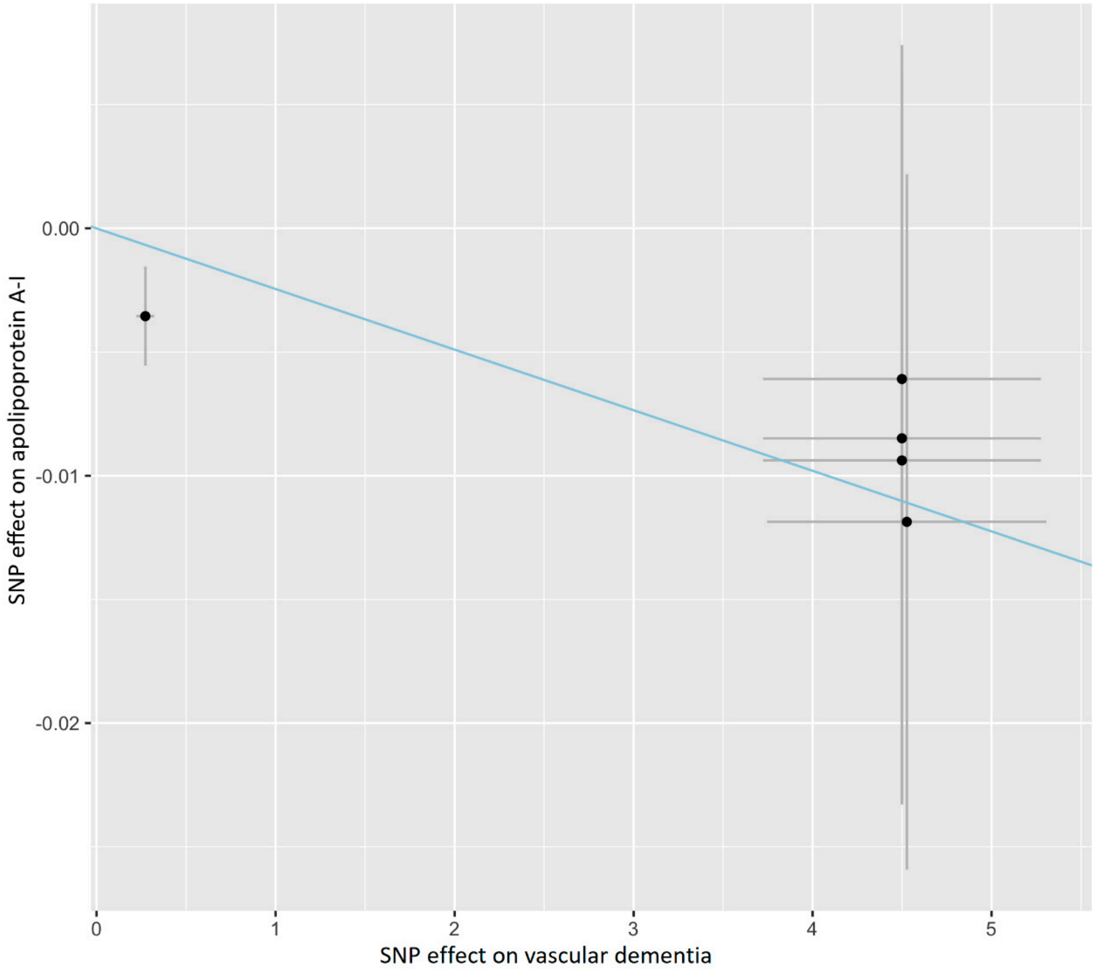

Supplementary Figure S3C. Scatter plot showing the association of the SNP effects on vascular dementia against the SNP effects on the apoA-I level.

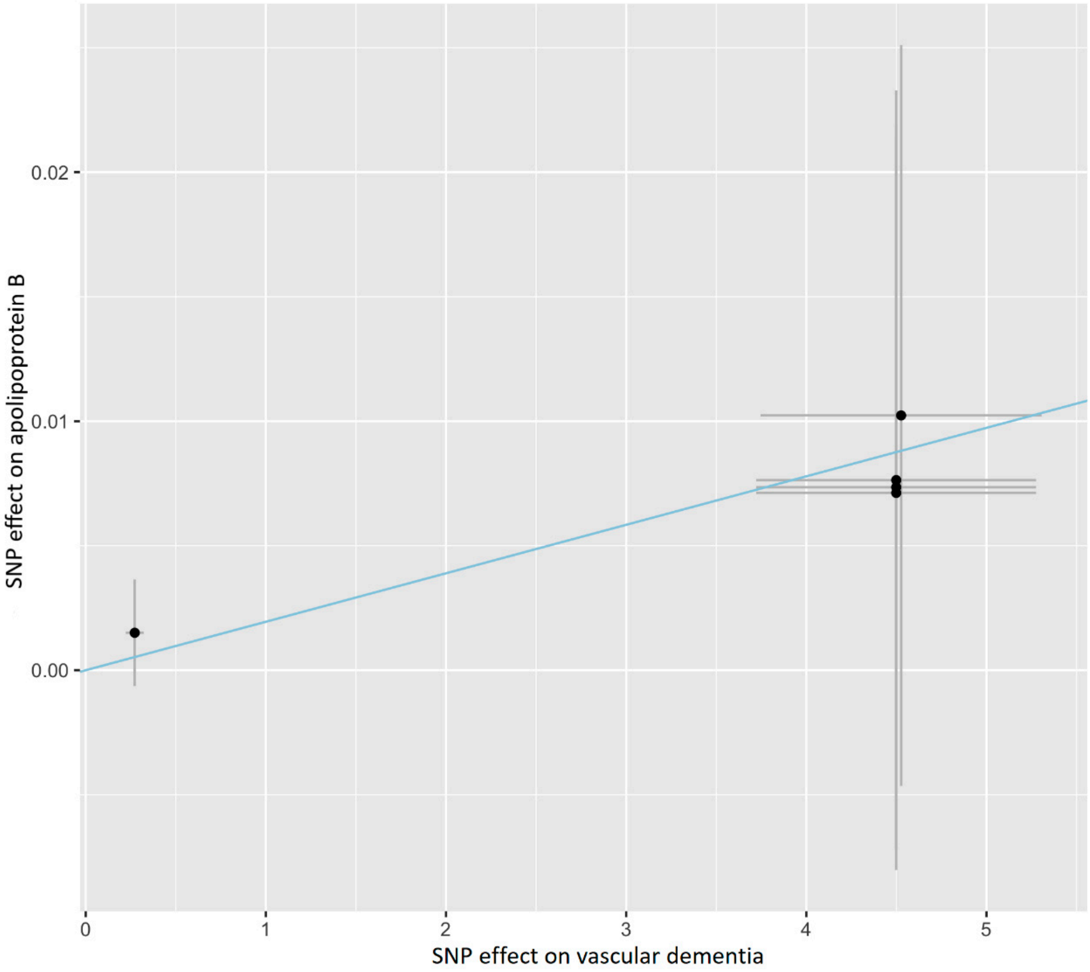

Supplementary Figure S3D. Scatter plot showing the association of the SNP effects on vascular dementia against the SNP effects on the apoB level.

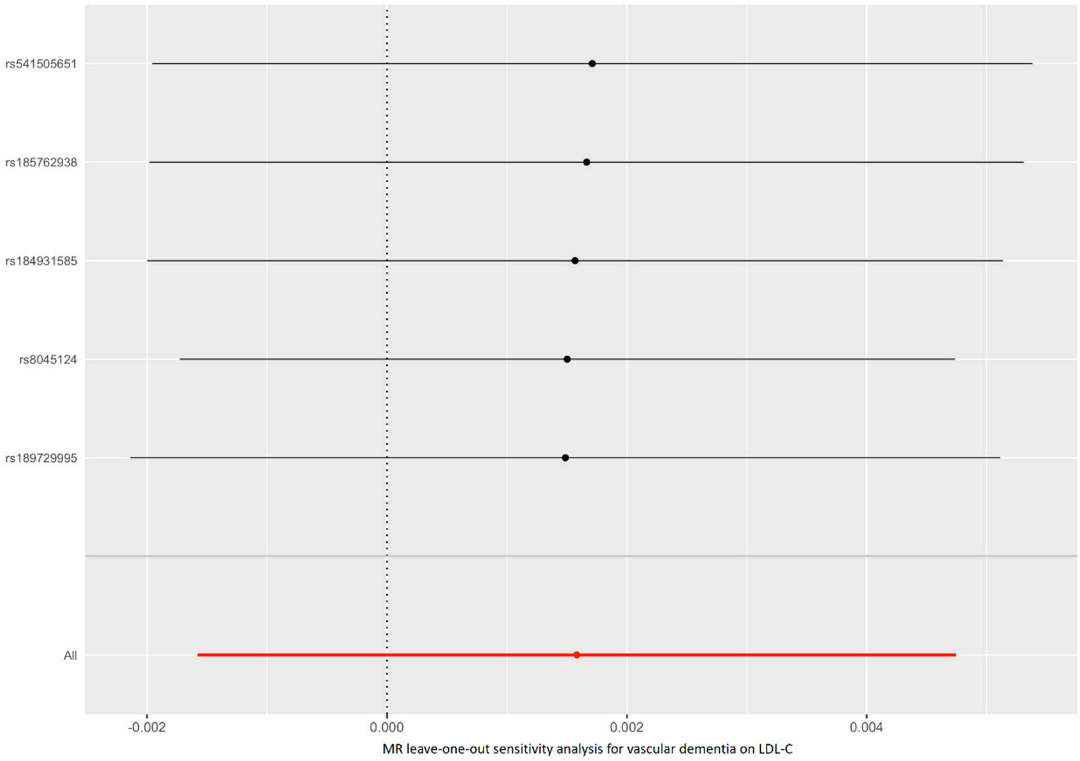

Supplementary Figure S4A. Leave-one-out permutation analysis of the causal association between vascular dementia and LDL-C level.

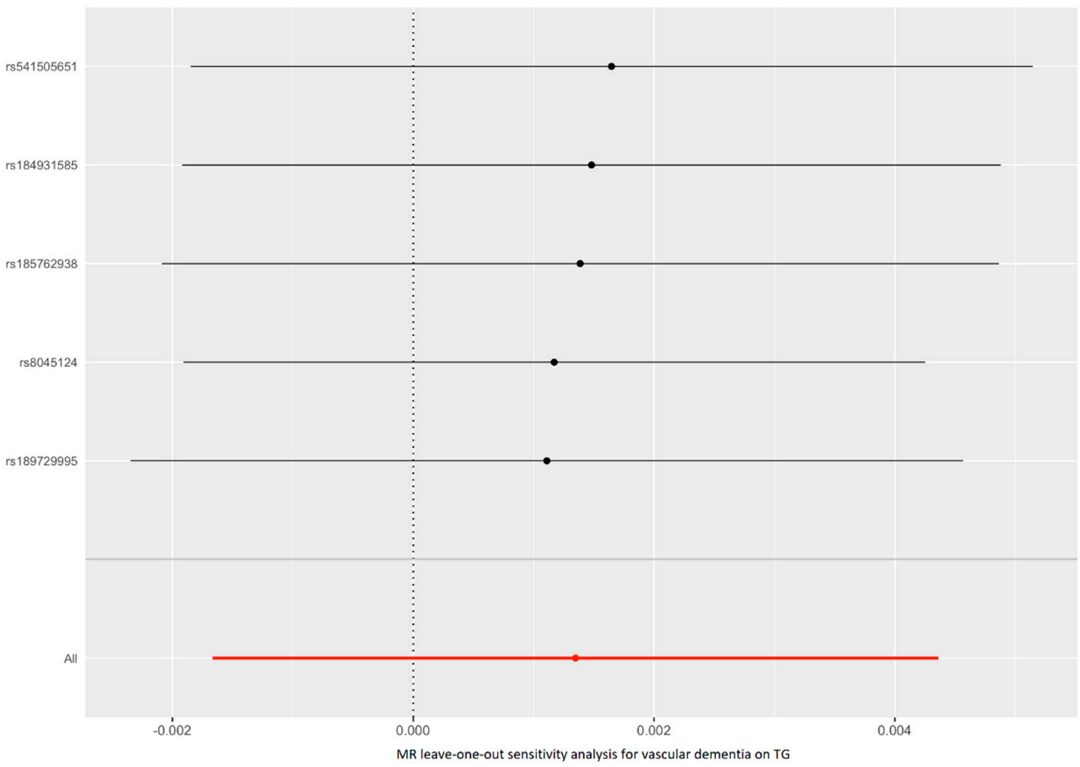

Supplementary Figure S4B. Leave-one-out permutation analysis of the causal association between vascular dementia and TG level.

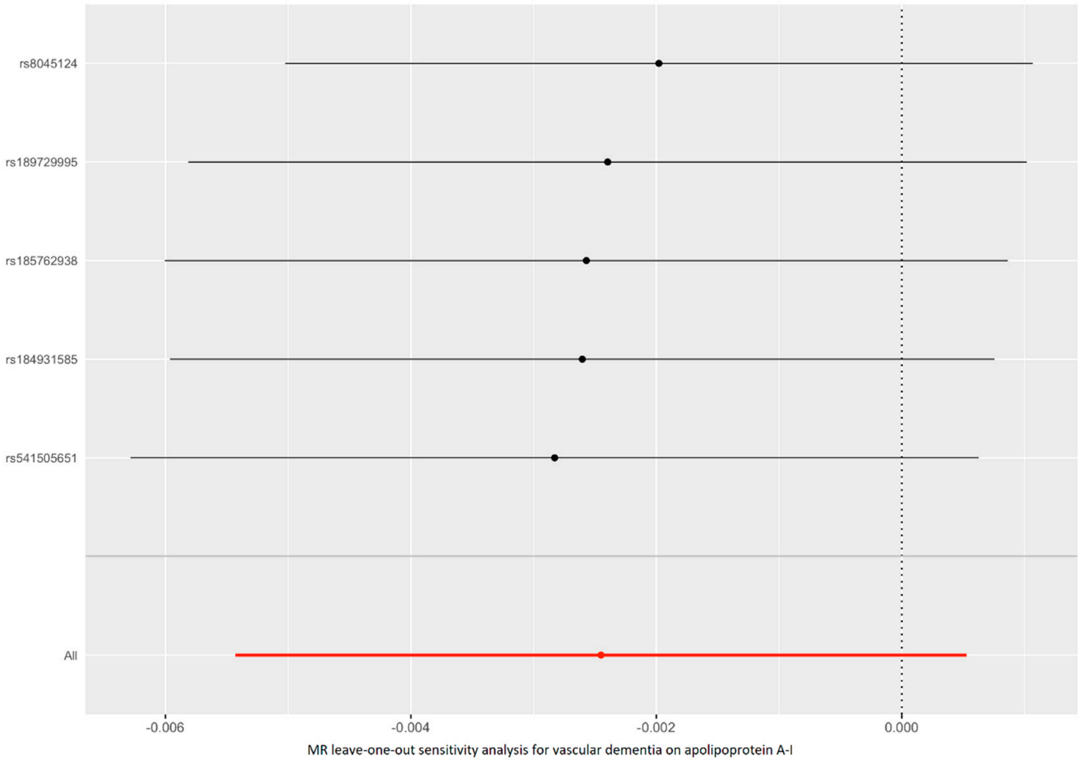

Supplementary Figure S4C. Leave-one-out permutation analysis of the causal association between vascular dementia and apoA-I level.

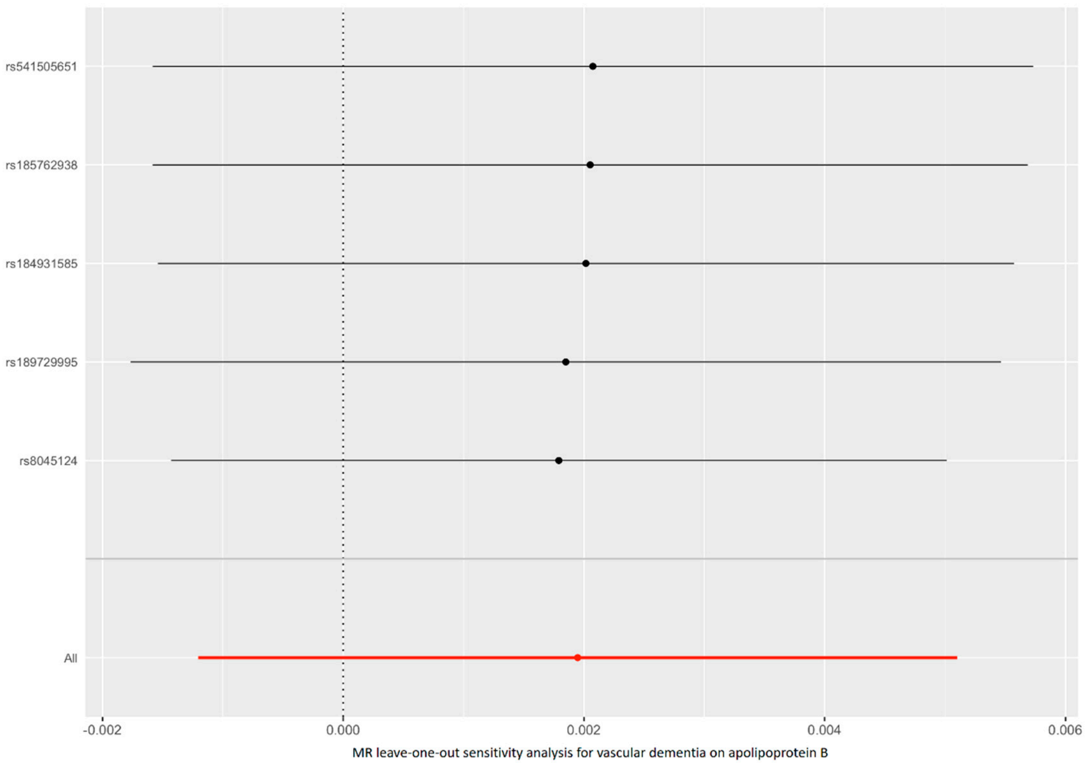

Supplementary Figure S4D. Leave-one-out permutation analysis of the causal association between vascular dementia and apoB level.

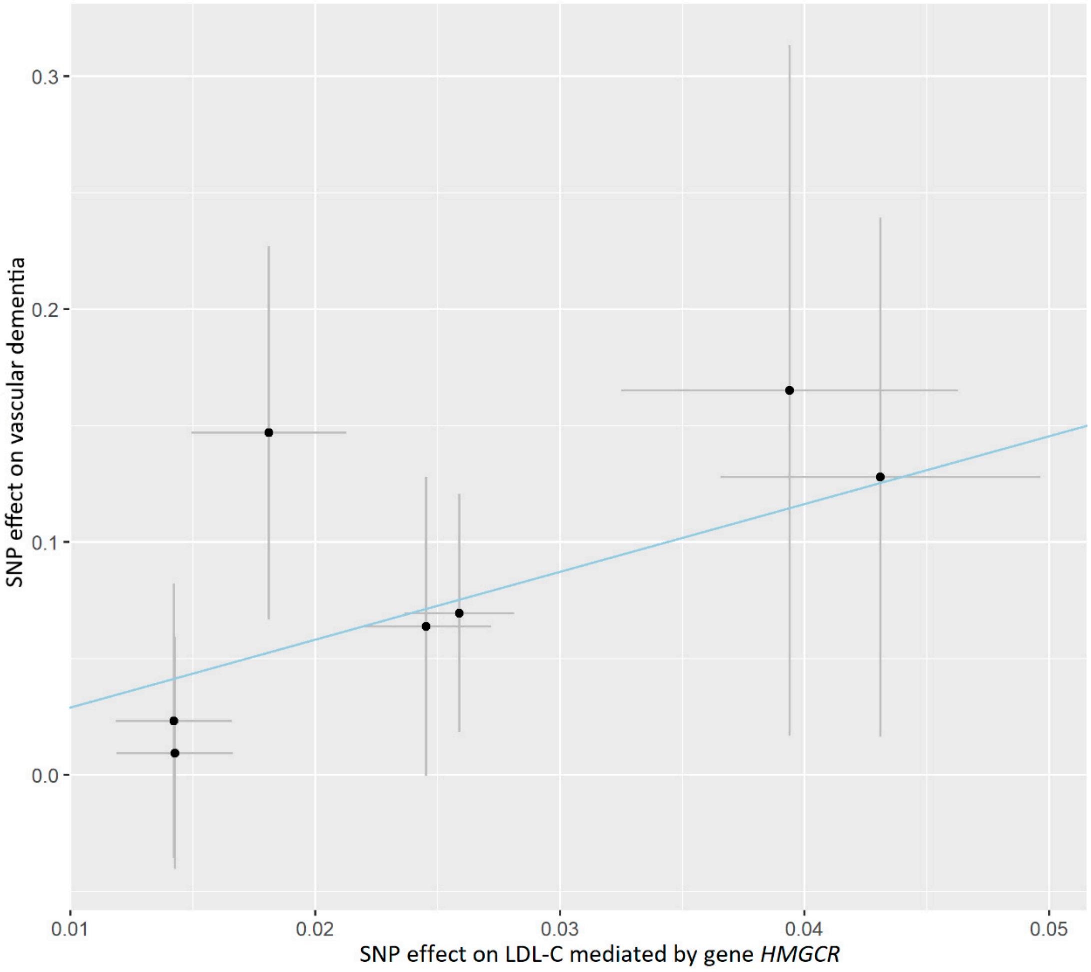

Supplementary Figure S5A. Scatter plot showing the association of the SNP effect on LDL-C level mediated by the *HMGCR* gene against the SNP effects on vascular dementia.

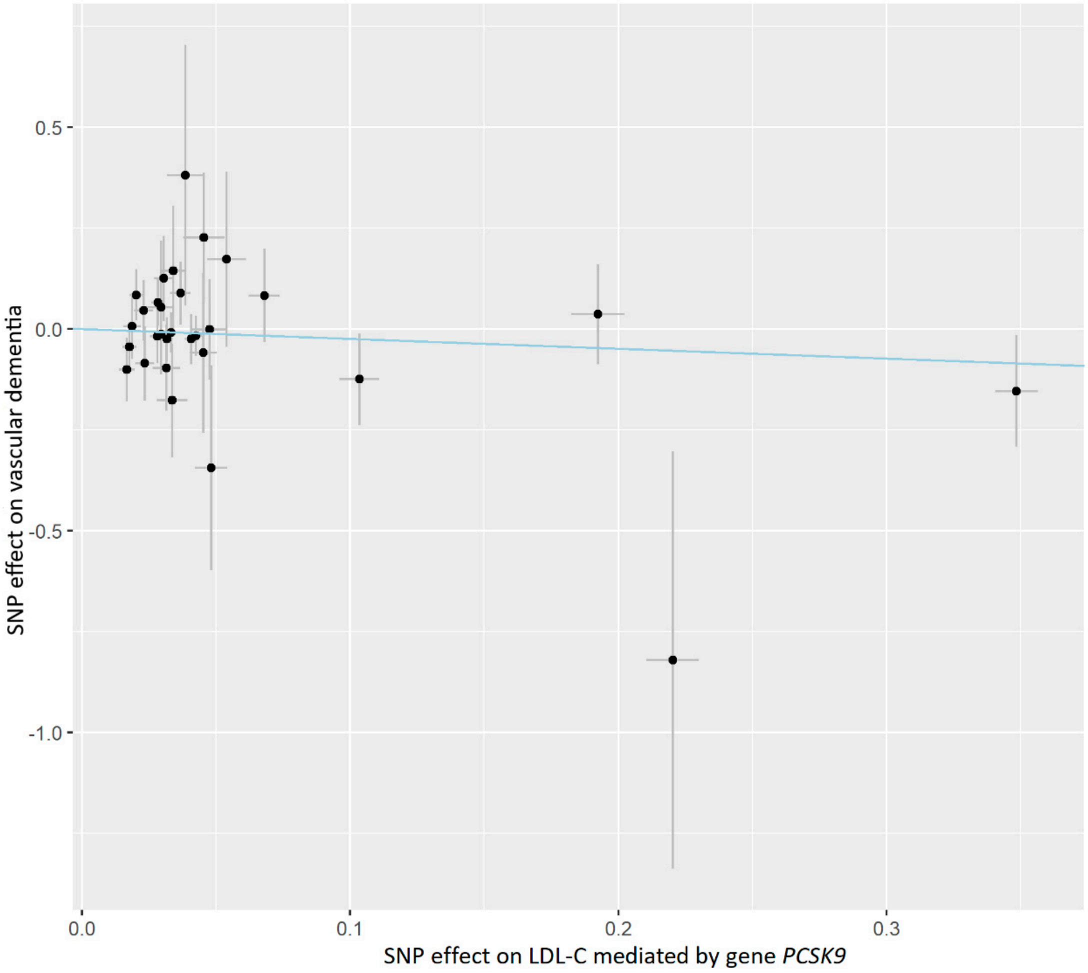

Supplementary Figure S5B. Scatter plot showing the association of the SNP effect on LDL-C level mediated by the *PCSK9* gene against the SNP effects on vascular dementia.

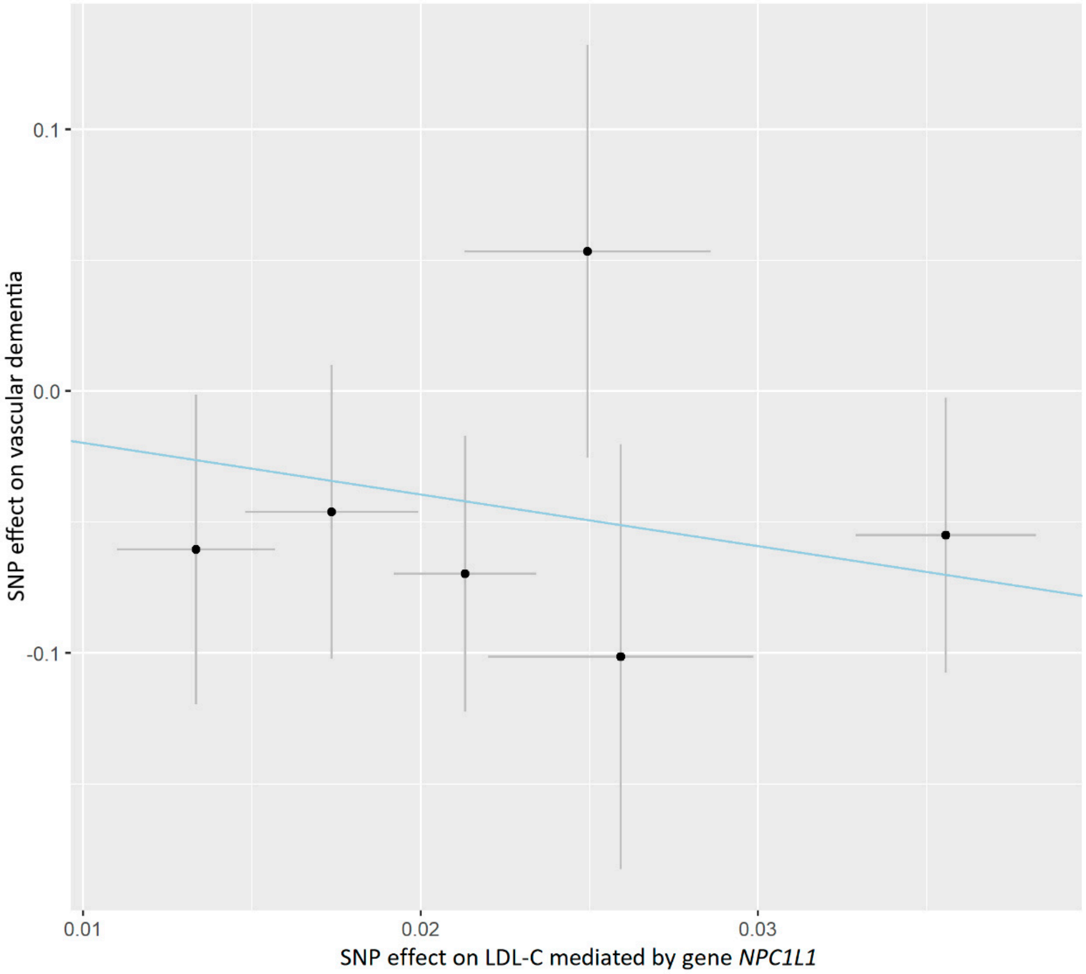

Supplementary Figure S5C. Scatter plot showing the association of the SNP effect on LDL-C level mediated by the *NPC1L1* gene against the SNP effects on vascular dementia.

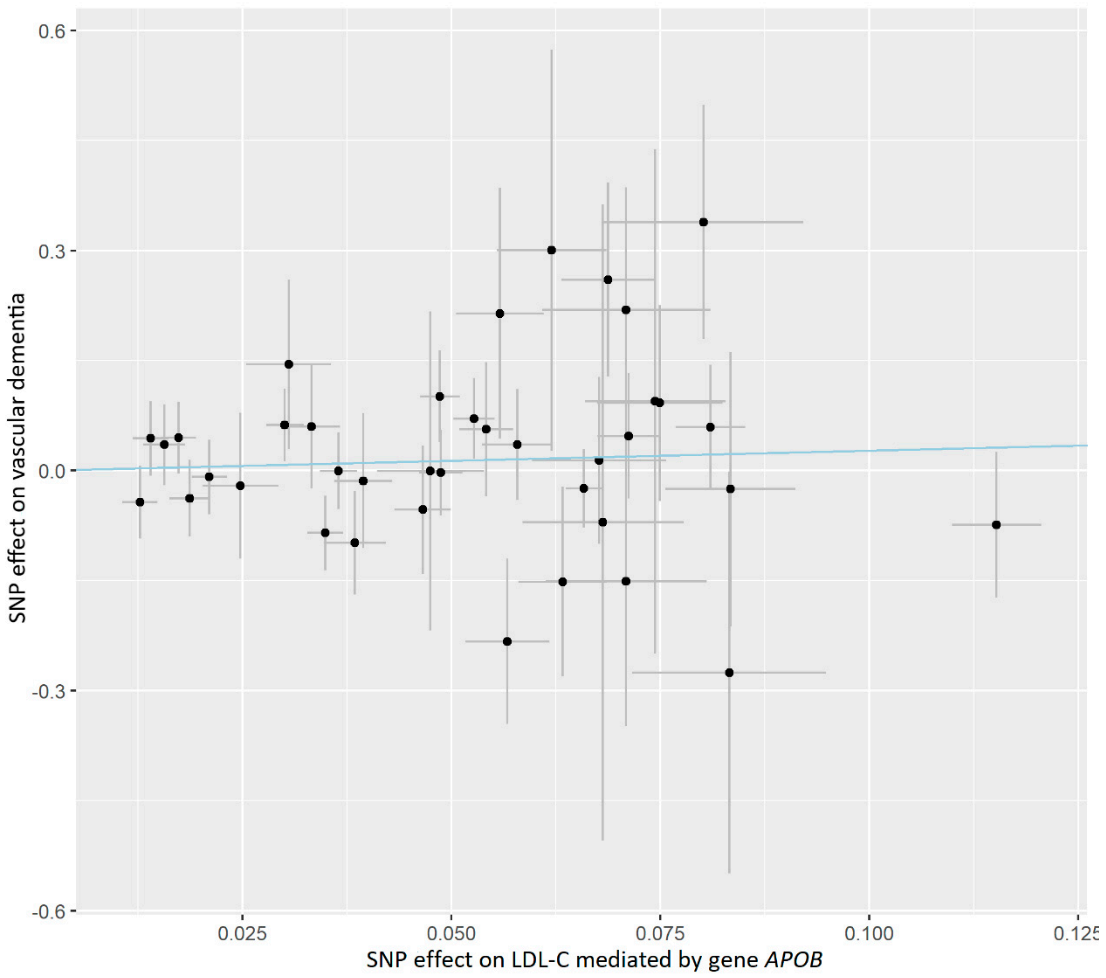

Supplementary Figure S5D. Scatter plot showing the association of the SNP effect on LDL-C level mediated by the *APOB* gene against the SNP effects on vascular dementia.

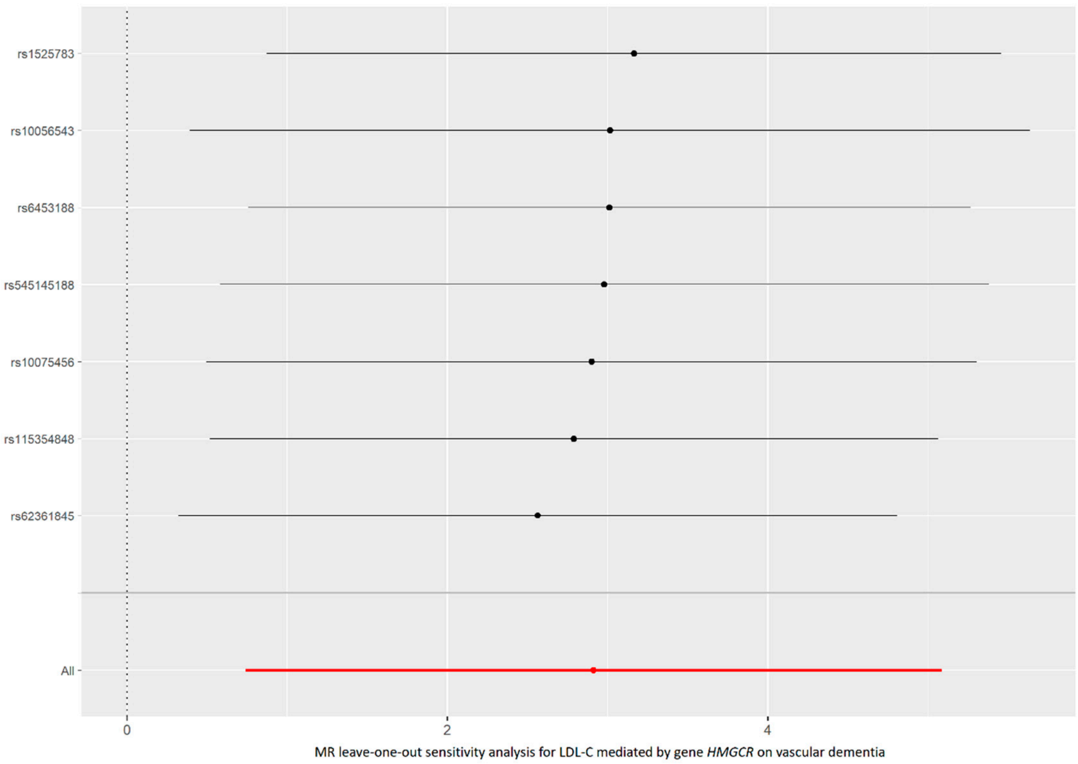

Supplementary Figure S6A. Leave-one-out permutation analysis of the causal association between LDL-C level mediated by the *HMGCR* gene and vascular dementia.

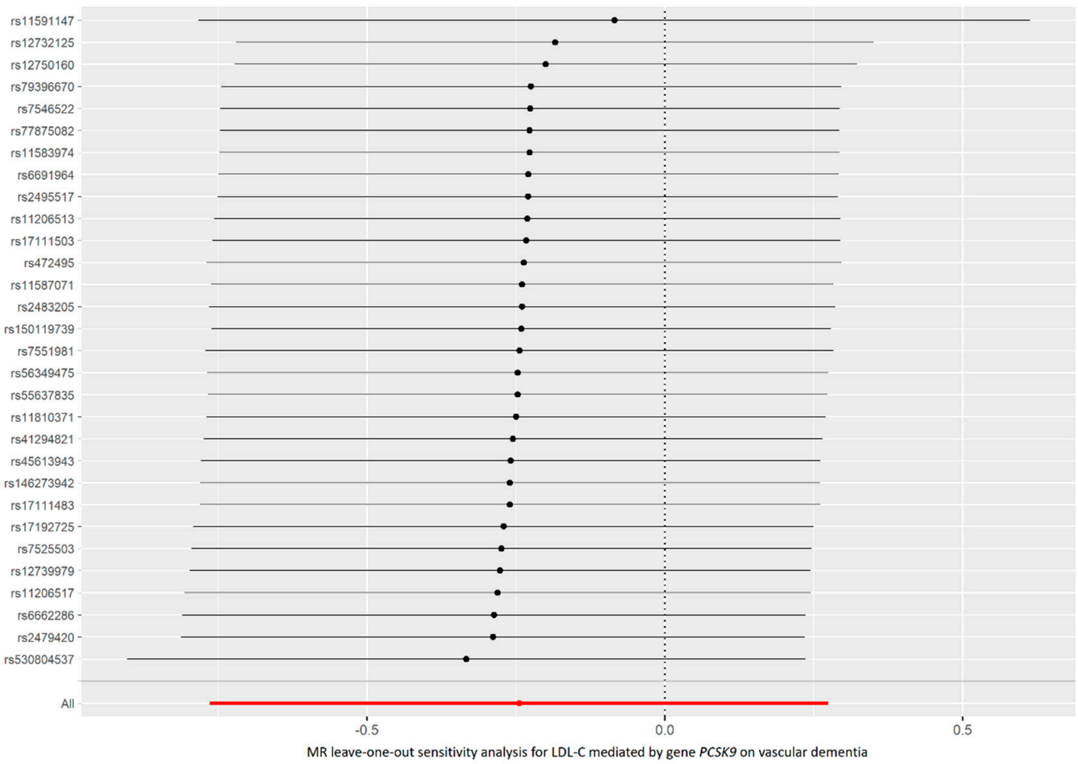

Supplementary Figure S6B. Leave-one-out permutation analysis of the causal association between LDL-C level mediated by the *PCSK9* gene and vascular dementia.

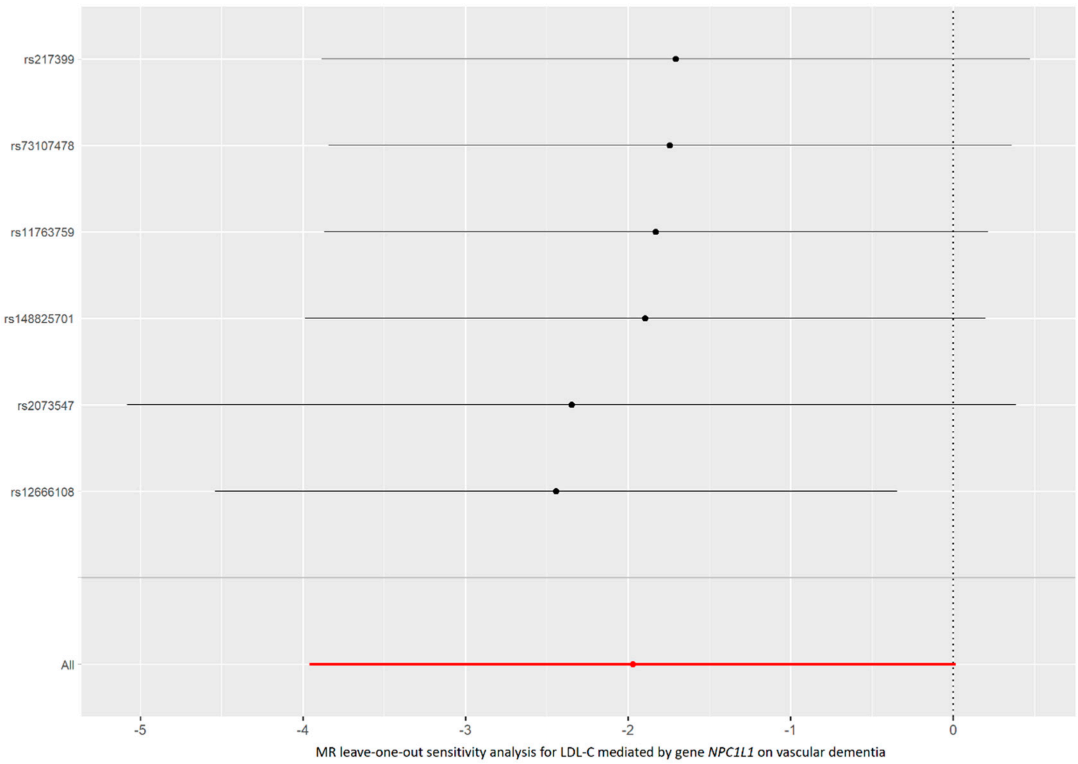

Supplementary Figure S6C. Leave-one-out permutation analysis of the causal association between LDL-C level mediated by the *NPC1L1* gene and vascular dementia.

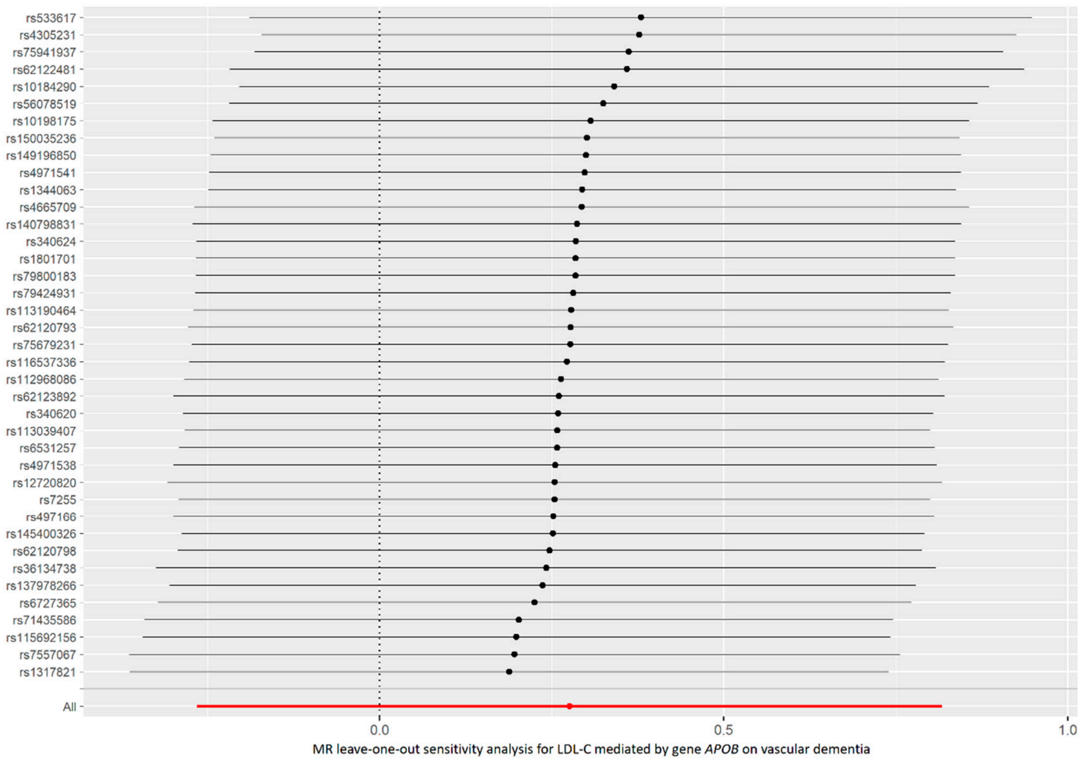

Supplementary Figure S6D. Leave-one-out permutation analysis of the causal association between LDL-C level mediated by the *APOB* gene and vascular dementia.
